# Advances in the multifunctionality of cell membrane-encapsulated nanoparticles in the treatment of colorectal cancer

**DOI:** 10.3389/fimmu.2025.1657722

**Published:** 2025-10-31

**Authors:** Zhengdong Qiu, Wenxuan Liu, Sheng Xu

**Affiliations:** ^1^ Department of Gastrointestinal, Hernia and Enteric Fistula Surgery, The People’s Hospital of Guangxi Zhuang Autonomous Region, Nanning, Guangxi, China; ^2^ Laboratory of General Surgery, Renmin Hospital of Wuhan University, Wuhan, Hubei, China

**Keywords:** CNPs, nanoparticles, immunity, colorectal cancer, immune microenvironment

## Abstract

Cell membrane-camouflaged nanoparticles (CNPs) have emerged as promising multifunctional platforms for colorectal cancer therapy, integrating drug delivery, immunomodulation, photothermal ablation, and anti-inflammatory effects. This review highlights recent advances in CNP-based strategies, emphasizing their unique capacity to enhance tumor-targeting specificity, potentiate immunotherapeutic efficacy, and overcome the limitations of conventional treatments. We summarize diverse approaches employing immune cell or tumor cell membrane coatings, as well as hybrid systems that combine CNPs with chemotherapy, metabolic modulation, or photothermal therapy. Accumulating evidence demonstrates that CNPs can effectively remodel the tumor immune microenvironment, increase the bioavailability of hydrophobic drugs, and promote synergistic therapeutic outcomes. Despite these encouraging results, clinical translation remains constrained by challenges in biodegradability, biosafety, large-scale manufacturing, and cost. Ongoing clinical trials are evaluating the safety and therapeutic potential of CNP-based nanomedicines. Overall, this review underscores the transformative role of CNPs as a next-generation platform for precision and personalized therapy in colorectal cancer.

## Introduction

Colorectal cancer (CRC) is among the most prevalent and lethal malignancies globally (1). Its pathogenesis is closely linked to various genomic alterations, such as chromosomal instability, microsatellite instability, and defects in CpG island methylation (2). Patients with early-stage CRC can be treated well with surgery, chemotherapy, or combination therapy, but there is a lack of effective treatment for patients with advanced CRC, especially for recurrent and metastatic CRC (3). Therapeutic challenges include tumor heterogeneity, immune cell dysfunction, immunosuppressive tumor microenvironment, and systemic immunotoxicity (4).

The application of nanomaterials in medicine is developing rapidly, and cell membrane-encapsulated nanoparticles (CNPs) in particular have attracted much attention due to their unique biointerfacial properties. These nanoparticles can mimic the functions of natural cells, such as “self”-labeling, interaction with the immune system, biotargeting, and localization to specific regions, leading to improved biocompatibility, reduced immunogenicity, immune escape, prolonged circulation time, and enhanced tumor targeting (5, 6). Treatments for colorectal cancer include surgery, chemotherapy, and immunotherapy. Surgery is the main treatment for early-stage CRC; chemotherapy and targeted therapy are commonly used for patients with advanced CRC, including oxaliplatin, fluorouracil, and irinotecan, as well as angiogenesis inhibitors and epidermal growth factor receptor inhibitors etc. (7). Immunotherapy, particularly immune checkpoint blockade (ICB) therapy and CAR T-cell therapy, offers new promise for the treatment of CRC, although response rates are currently low (8).

CNPs provide distinct advantages in the therapeutic management of colorectal cancer. For instance, erythrocyte membrane-camouflaged nanoparticles can prolong systemic circulation and enhance tumor accumulation by reducing immune clearance and improving vascular retention. In contrast, platelet membrane-coated nanoparticles possess intrinsic affinity for subendothelial matrices, thrombotic sites, and activated endothelial cells, thereby enabling precise targeting across multiple stages of tumor progression (9). CNPs enhance therapeutic efficacy by mimicking the function of natural cells, improving the efficiency and targeting of drug delivery while reducing clearance by the immune system (10).

**Figure 1:**
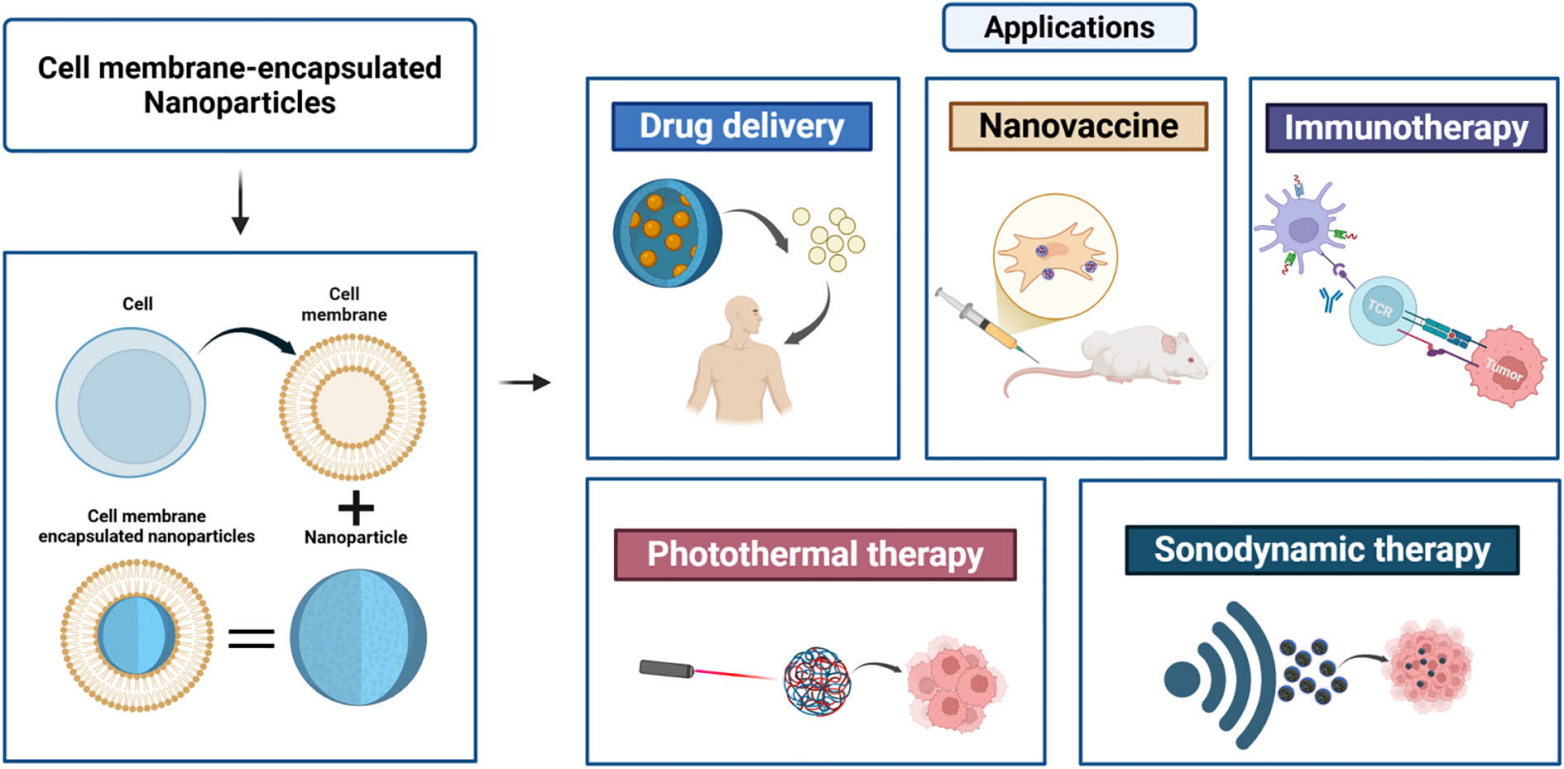
Preparation of cell membrane-encapsulated nanoparticles and their applications. Cell membrane-encapsulated nanoparticles are a cutting-edge biotechnology platform that forms a “bionic” structure by enveloping cell membranes around nanoparticles. This approach demonstrates broad application potential across multiple advanced therapeutic fields, including drug delivery, vaccine development, immunotherapy, and physical therapies (light, sound).

The treatment of colorectal cancer is gradually shifting from traditional surgery and chemotherapy to more precise immunotherapy and nanomedicine. This review explores the development of CNPs for the treatment of colorectal cancer, providing a comprehensive analysis of their applications in anticancer drug delivery, photothermal therapy, and immunotherapy. Future considerations for translating promising CNP platforms into the clinic are also discussed (**Figure 1**).

## Status and classification of CNPs

CNPs, as an innovative nanocarrier, form a core–shell structure with cell-mimicking properties by covering natural cell membranes on a synthetic core (11). Due to their unique membrane structure and surface antigens, CNPs have significant advantages in drug delivery, photothermal therapy, and immunotherapy (12). The manufacturing process of CNPs involves the use of a variety of cell membrane materials, such as red blood cells, immune cells, and cancer cell membranes, each of which gives CNPs unique properties that enable them to play a key role in cancer therapy (9, 10, 13).

Red blood cell membrane-coated nanoparticles (RBCM) show significant advantages in tumor therapy due to their long-lasting properties, which prolong the circulation time in the body and thus increase the exposure to tumors (9). These nanoparticles play an important role in phototherapy, especially photothermal therapy (PTT) and photodynamic therapy (PDT), e.g., red blood cell membrane nanovesicles (RMNVs) conjugated with biomimetic black phosphorus quantum dots (BPQDs) effectively induce apoptosis of cancer cells and activate tumor-specific immune responses under near-infrared laser irradiation (14). In addition, RBCM has been used for efficient delivery of anticancer drugs (15). In immunotherapy, a novel immunotherapeutic system (Lmo@RBC) was constructed by wrapping erythrocyte membranes around bacteria, selectively deleting virulence factors. Lmo@RBC synergistically triggers tumor cell pyroptosis by upregulating the expression of the pore-forming protein GSDMC and activating ROS-induced activation of cysteine-containing aspartic acid protein hydrolase 8 (caspase-8), which is effective in inducing an antitumor immune response (16). The above demonstrates the potential of erythrocyte membrane-encapsulated nanoparticles to enhance the efficacy of tumor therapy and immune response.

Immune cell membrane-encapsulated nanomaterials play multiple roles in tumor therapy. They are able to present tumor antigens directly to the immune system to activate antitumor immune responses and to construct tumor vaccines to enhance the maturation and activation of dendritic cells to stimulate specific immune responses (17, 18). In addition, these nanomaterials can improve the tumor microenvironment, e.g., by enhancing antitumor immunity through reprogramming tumor-associated macrophages and alleviating tumor hypoxia (18). T lymphocyte membrane-encapsulated nanomaterials have also been used to activate T lymphocytes, further enhancing the efficacy of cancer immunotherapy (19). Macrophage membrane-coated nanoparticles, such as silica nanoparticles and near-infrared imaging probe-loaded gold nanoparticles, not only enhance drug delivery efficiency and anticancer effects but also increase tumor tissue accumulation under near-infrared laser irradiation, generate local heat to effectively inhibit tumor growth, and achieve selective ablation of cancer cells in the region of thermal irradiation (20). These applications demonstrate the potential and versatility of immune-cell membrane-encapsulated nanomaterials in tumor therapy.

Cancer cell membrane-encapsulated nanoparticles show potential for multifaceted applications in the field of tumor therapy. By co-loading photosensitizers and Toll-like receptor 7 agonists, they are able to enhance the efficacy of combined tumor immunotherapy by generating reactive oxygen species (ROS) to kill tumor cells in combination with photodynamic therapy and activating the host’s antitumor immune response to remove residual tumor cells (21). In addition, these nanoparticles enhance the bioavailability of poorly water-soluble drugs, increase selective drug accumulation in tumors, and improve therapeutic efficacy while reducing systemic toxicity (22). They also incorporate inducers of immunogenic cell death (ICD), enhance ICD in tumor cells, facilitate the release of tumor-associated antigens and damage-associated molecular patterns, thereby stimulating antitumor immune responses (23). In the construction of tumor vaccines, these nanoparticles enhance the maturation and activation of dendritic cells to stimulate specific antitumor immune responses and efficiently target and stimulate the maturation of DCs by modifying the TLR9 agonist, CpG oligonucleotides, and aptamers on tumor cell membrane vesicles to generate long-lasting antitumor immune responses (24).

**Figure 2:**
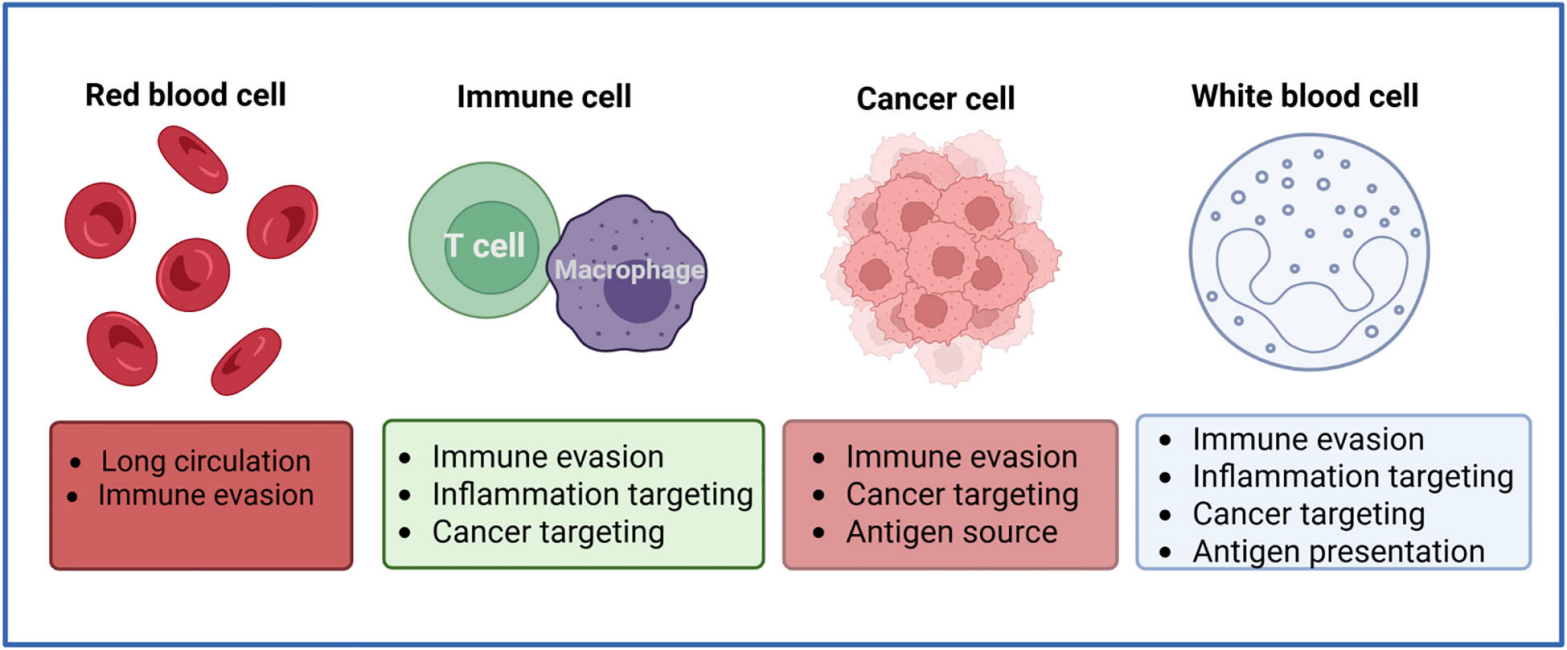
Sources of common cell membranes used for preparing cell membrane-encapsulated nanoparticles. Cell membrane-coated nanoparticles can be produced using cell membrane materials from red blood cells, immune cells, cancer cells, and leukocytes. Each type of membrane coating confers specific properties that can be used in anticancer applications.

The abovementioned powerful potential of these cell membrane-encapsulated nanoparticles to improve therapeutic efficacy and to activate and enhance the body’s antitumor immune response provides a new strategy for achieving personalized and precise tumor immunotherapy (**Figure 2**).

## CNPs in colorectal cancer treatment

### Drug delivery

Inflammatory bowel disease (IBD) is a chronic inflammatory disorder of the gastrointestinal tract and is closely linked to an elevated risk of colorectal cancer. Research has demonstrated that individuals with IBD have a significantly higher risk of developing CRC (25). A team has developed a bionic vesicle (SLKs) that specifically targets leukocytes and used it to treat a mouse model of IBD. Experimental results showed that this treatment significantly reduced the inflammatory response and promoted the repair of intestinal epithelial tissue (26). Duan et al. developed an oral formulation of macrophage membrane-encapsulated nanoparticles (cp-MΦ-NPs) that bind and neutralize pro-inflammatory cytokines, significantly reducing the severity of IBD (27). Studies have shown that liposome-coated nanoparticles for the delivery of the inflammatory factor interleukin (IL)-12 (PLE-IL-12-NPs) and PLE-IL-12-NPs are able to release active IL-12 *in vivo* and trigger an immune response to inhibit the growth of colorectal cancer (28). In addition to interleukin inflammatory factor, tumor necrosis factor-α-related apoptosis-inducing ligand (TRAIL) is also highly expressed in colorectal tumor cells, and studies have shown that the combination of the chemotherapeutic agent oxaliplatin with immunohybrid nanoparticles (OIHNPs) enables synergistic delivery of oxaliplatin and anti-TRAIL, leading to therapeutic inhibition of colorectal cancer tumor models (29). Studies have shown that the transient delivery of miR-200 via oral administration of miR-200-loaded lipid nanoparticles (LNPs) restores intestinal stem cells (ISCs) and enhances intestinal regeneration in mice following acute injury (30).

In recent times, metabolic reprogramming has emerged as a significant hallmark of cancerous cells, characterized by elevated glycolysis and augmented lactic acid fermentation. As a result, the inhibition of glycolysis is considered a promising therapeutic strategy for colorectal cancer. Glycolysis can be classified into two distinct categories: aerobic glycolysis and anaerobic glycolysis, which may or may not involve the use of oxygen. 2-Deoxy-d-glucose (2DG) is capable of eliminating the energy source of glucose glycolysis by inhibiting it, which ultimately leads to the death of cancer cells due to starvation. Wu et al. constructed a multifunctional nanoplatform containing a core of rare-earth doped nanoparticles (LnNPs) by combining mesoporous silica shells containing 2DG and the photosensitizer chlorinated e6 (Ce6) in mesoporous channels. In combination with photodynamic therapy, which disrupted mitochondrial function and enhanced the efficacy of 2DG by inhibiting the expression of hexokinase 2 (HK2) and lactate dehydrogenase, thereby inhibiting glucose metabolic reprogramming and increasing the efficiency of starvation therapy to inhibit colorectal cancer tumor growth (31). The inhibition of glucose supply using glucose oxidase (GO*_x_*) offers an alternative therapeutic strategy for tumor starvation (32). However, the *in vivo* application of GO*_x_*-based starvation therapy is significantly hindered by poor GO*_x_* delivery efficiency and self-limiting therapeutic effects. To address these challenges, Zhang et al. developed a nanoplatform that encapsulates GO*_x_* and tirazamine (TPZ) within erythrocyte-membrane-masked metal-organic framework (MOF) nanoparticles (TGZ@eM). This platform facilitates efficient GO*_x_* delivery to tumor cells, effectively depleting endogenous glucose and oxygen to induce tumor cell starvation. Notably, the hypoxia induced by this approach further activates the TPZ released from the nanoplatforms in the acidic lysosomal/endosomal environment, thereby enhancing the therapeutic efficacy against colon cancer (33). A study has developed a photosynthetic leaf-inspired abiotic/biotic nanolike vesicle (PLANT) system that employs its photosensitivity and capacity to catalyze the decomposition of hydrogen peroxide to enhance the oxygen levels of tumor cells and induce apoptosis, which in turn inhibits colorectal cancer cell growth (34). Hexokinase (HK) is the rate-limiting enzyme in the initial step of glycolysis. Benserazide, a selective inhibitor of HK2, has been shown to specifically bind to HK2 and markedly inhibit its enzymatic activity *in vitro (* 35). In a study conducted by Li et al., benserazide nanoparticles were prepared and shown to demonstrate inhibition in a mouse model of colorectal cancer. Furthermore, the nanoparticles were demonstrated to be more efficacious than conventional treatment (36). The role of hydrogen peroxide (H_2_O_2_) in colorectal cancer has been the subject of numerous studies, which have revealed that it exerts a multifaceted influence on tumor cells. In addition to promoting the proliferation and metastasis of tumor cells, H_2_O_2_ has also been identified as a signaling molecule that induces apoptosis (37). In a single colorectal cancer Caco-2 cell, Wang et al. accurately measured the H_2_O_2_ gradient from extracellular to intracellular using highly sensitive platinum-functionalized nanomaterials in combination with noncontact hopping probe mode scanning ion conductivity microscopy (hPICM), a technique that allows for the measurement of ionic currents with high sensitivity and spatial resolution (38).

It has been demonstrated that resveratrol is an effective inhibitor of the mechanism of colon cancer cell growth through a ROS-dependent iron-concentration pathway (39). Zhang et al. developed an innovative biomimetic nanocarrier system, resveratrol (RSV)-NPs@RBCm, which achieves efficient encapsulation of RSV through the encapsulation of poly(ϵ-caprolactone)-poly(ethylene glycol) nanoparticles within erythrocyte membranes. The nanoparticles were prepared using poly(ϵ-caprolactone)-poly(ethylene glycol) (PCL-PEG) and erythrocyte membranes. When combined with the tumor-penetrating peptide, iRGD, RSV-NPs@RBCm demonstrated a significant enhancement in tumor penetration ability, thereby facilitating the treatment of colorectal cancer (40). Zeng et al. employed activated neutrophil membrane-encapsulated nanoparticles (aNEM NPs) as nanodecoys, which demonstrated a notable neutralizing impact and were capable of effectively inhibiting chemokine-mediated recruitment of neutrophils to the primary tumor, thereby mediating tumor growth (41). The hydrophobicity of certain pharmaceutical agents may present a challenge to their potential clinical utilization (42). The nanoprecipitation method, as employed by Zhang et al., was used to prepare ursolic acid-containing nanoparticles (UA-NPs). This resulted in enhanced apoptosis through the stronger inhibition of COX-2 and activation of cysteine-aspartic enzyme 3 when compared to free UA-NPs. Additionally, the nanoparticles demonstrated superior penetration of cell membranes (43). It has been demonstrated that the co-coating of apigenin and the chemotherapeutic drug 5-FU in multifunctional nanomaterials can result in a synergistic effect, thereby significantly enhancing the efficacy of chemotherapy *in vivo* in patients with CRC (44).

In conclusion, CNPs demonstrate considerable potential and significance in multiple pivotal domains. They can effectively modulate the inflammatory response, intervene against the glycolytic process in colorectal cancer, and achieve precise delivery of chemotherapeutic drugs. Additionally, they can reduce the hydrophobicity of drugs, enhance their bioavailability, and prolong the half-life of drugs *in vivo*. These advantages render the nanocarrier system a highly efficacious instrument for enhancing therapeutic outcomes and patients’ quality of life.

### Nanomaterials combined with immunotherapy and PTT

Despite the extensive development of nanoparticle-based tumor therapeutic strategies, the therapeutic efficacy remains constrained by the inadequate accumulation of nanoparticles in tumor tissues and the suboptimal antitumor effect of single treatment modalities (45). The distinctive characteristics of nanomaterials and their collaborative effects with alternative therapeutic modalities have prompted the majority of researchers to develop treatments that are more efficacious than traditional therapies, thereby offering patients a more comprehensive and precise range of treatment options.

CD47 is markedly expressed in colorectal cancer tissues, and its elevated expression is correlated with a poor prognosis. It triggers immunosuppressive signaling pathways by binding to signal regulatory protein α (SIRPα) on macrophages, thereby enabling cancer cells to evade immune surveillance and clearance (46). Wang et al. constructed a dual-membrane-camouflaged miRNA21 antagonist delivery nanoplatform (M@NPs/miR21), which was self-assembled from ZnO/miRNA21 antagonist nanoparticles (NPs/miR21) and the membranes of MC38 tumor cells (M38) and macrophage cells (Ma) by ultrasonication. The delivery of miR21 antagonists to tumor tissues is effectively facilitated, resulting in the inhibition of miR21 expression, induction of tumor cell apoptosis, regulation of Bcl2 and Ki67 expression, and subsequent inhibition of colorectal tumor growth and metastasis (47). The nanoplatform retains the antitumor effect of ZnO nanoparticles while also exhibiting CD47 protein-mediated immune escape signaling and galectin-3 protein-mediated tumor cell aggregation. Professor Zhiyong Qian’s team developed the MPB-3BP@CM NPs cell membrane biomimetic nanomedicine platform, which employs microporous Prussian blue nanoparticles (MPB NPs) as a carrier for photothermal sensitizers and 3-bromopyruvic acid (3BP) encapsulated in cell membranes that express a high-affinity signal for protein variant SIRPα. The MPB-3BP@CM NPs could prolong the blood circulation time, effectively target colorectal cancer cells CD47, and inhibit colorectal cancer growth by blocking the CD47-SIRPα interaction and promoting the polarization of tumor-associated macrophages (TAMs) towards the M1 phenotype. Furthermore, the combination of MPB NPs-mediated photothermal therapy enhances the therapeutic efficacy against tumors (48). It has been demonstrated that red blood cells possess intrinsic characteristics that result in a prolonged cycle time (9). In order to obtain Cyp-MNC@RBC with significantly improved physiological stability in comparison to bare MNC, Wang et al. encapsulated superparamagnetic nanoclusters (MNC) with erythrocyte membranes. Furthermore, the researchers loaded the Cyp-MNC@RBC on near-infrared (NIR) carriers, which resulted in a significant increase in both NIR absorbance and photothermal conversion efficiency (49).

In the tumor microenvironment, the conventional mode of tumor cell death frequently lacks immunogenicity, which enables tumors to evade detection by the immune system. However, by inducing immunogenic cell death, tumor cells can release tumor-associated antigens and cytokines, which are molecular signals that recruit antigen-presenting cells and effector CD4^+^ and CD8^+^ T cells. This, in turn, activates both natural and adaptive immune responses, which enhances the effectiveness of antitumor immunotherapy (8). A team of researchers has developed a multifunctional biomimetic nanoplatform (Fe_3_O_4_@PDA@CaCO_3_-ICG@CM) based on magnetic dopamine (PDA) modified with CaCO_3_ containing indocyanine green (ICG). This nanomaterial has been shown to effectively scavenge programmed death ligand 1 (PD-L1) and transforming growth factor-beta (TGF-β), in addition to other functions. Furthermore, it induces apoptosis of CT26 cells by combining with photothermal therapy. This process can induce ICD, which activates the maturation of dendritic cells and subsequently promotes the activation of CD4^+^ and CD8^+^ T cells, thereby inhibiting the growth of colorectal cancer cells (50). In a novel approach, Li et al. developed a nanoplatform that employs leukocyte membranes encapsulated with glycyrrhizinic acids (GCMNPs) as an efficacious inducer of cellular immunogenic death. This design not only elicited a systemic immune response in CRC mice but also markedly reduced toxicity to the CRC (51).

Immune checkpoint blockade therapies targeting programmed death 1 (PD-1) or its primary ligand, PD-L1, have achieved remarkable success in the treatment of a wide range of tumors, including colorectal cancer. Nevertheless, the efficacy of PD-1/PD-L1 inhibitors is constrained in certain colorectal cancers characterized by an immunosuppressive tumor microenvironment, such as a low degree of immune cell infiltration (8). Xiao et al. developed anti-PD-L1 functionalized biomimetic dopamine-modified gold nanostar nanoparticles (PDA/Gold nanostar (GNS)@aPD-L1 NPs), which demonstrated considerable efficacy in inhibiting tumor growth and reducing the number of immunosuppressive cells. The maturation of dendritic cells is enhanced, and the infiltration of CD8^+^ T cells is increased, leading to prolonged overall survival. Additionally, PDA-GNS-mediated local photothermal ablation of tumors promotes the release of tumor-associated antigens, thereby activating the antitumor immune response. Simultaneously, photothermal therapy suppresses colorectal cancer growth by increasing tumor permeability and facilitating immune cell infiltration (52). Liu et al. designed a multifunctional nanomaterial by incorporating oxaliplatin (OXA) and ICG into hyaluronic acid (HA)-modified metal-organic framework MIL-100 nanoparticles, resulting in the development of multifunctional nanoparticles (OIMH NPs). These nanomaterials were shown to induce immunogenic cell death and enhance immune cell infiltration within the tumor microenvironment, thereby improving the efficacy of immune checkpoint blockade therapy (53). Wang et al. successfully constructed a novel nanocomposite, a hollow gold nanocage nanocomposite (GNC-Gal@CMaP), which demonstrated selective targeting of colon cancer cells and effective accumulation in the tumor microenvironment. The material effectively integrates PTT, a PD-L1 antibody, and a TGF-β inhibitor (galunisertib), subsequently enhancing the antitumor efficacy of an anti-PD-L1 antibody and galunisertib through the activation of antigen-presenting cells, which in turn trigger tumor-specific effector T cells (54).

The aforementioned findings indicate that multifunctional nanomaterials can effectively enhance the targeting and efficacy of photothermal therapy PTT, thereby potentiating the anticolorectal cancer tumor effect when employed in conjunction with immunotherapy.

### Imaging technology

**Figure 3:**
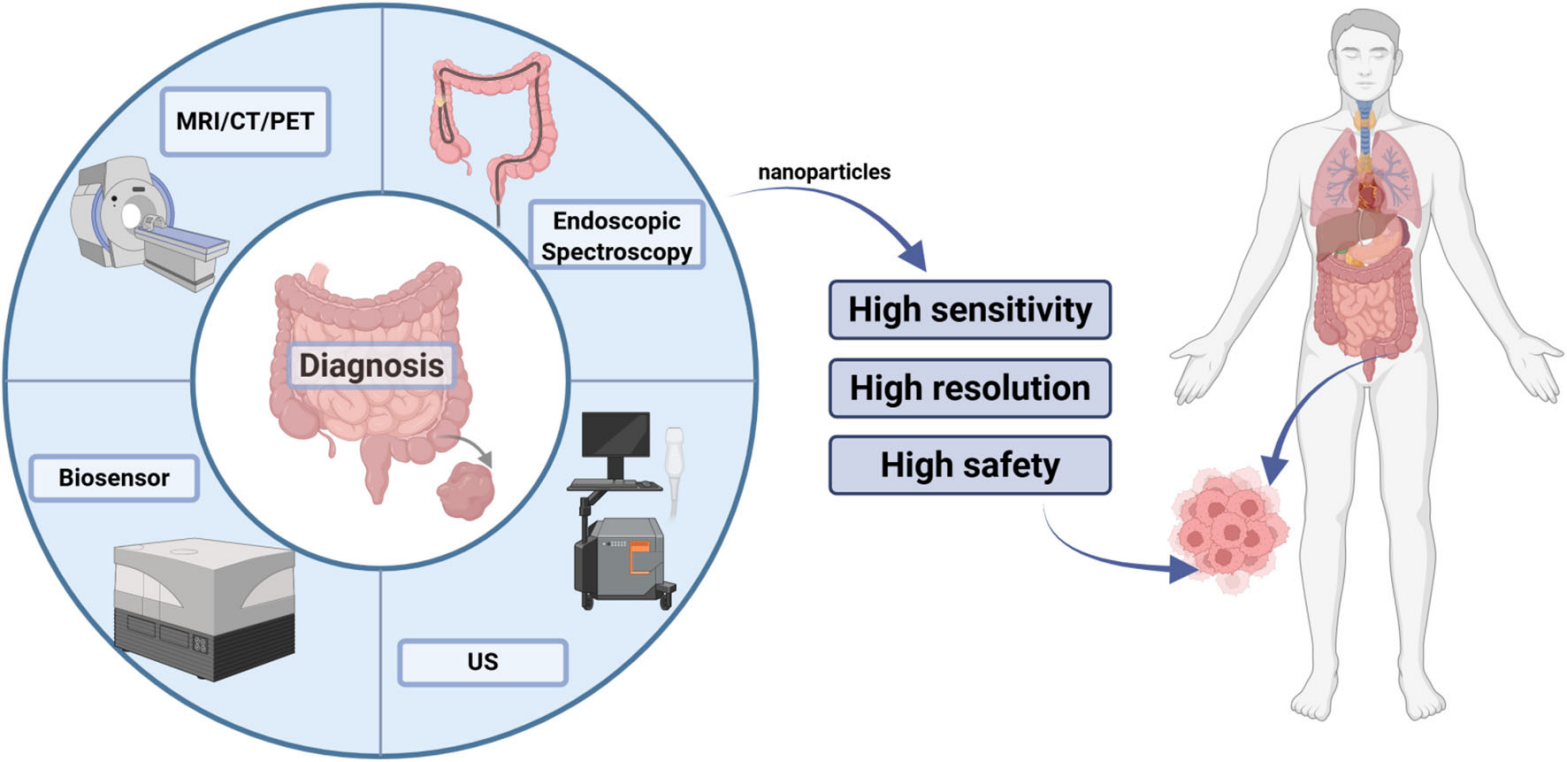
Role of nanomaterials in imaging technology. In colorectal cancer imaging, nanoparticle-based targeted contrast agents integrate seamlessly with endoscopy, CT, and MRI to boost sensitivity down to submillimetric early lesions, sharpen resolution for accurate staging, and maintain exemplary biocompatibility to ensure patient safety.

The suppressor of cytokine signaling 1 (SOCS-1) has been demonstrated to influence the development of colorectal cancer and the process of immune escape by regulating cytokine signaling (55). Guthula et al. have developed an innovative fiber optic nanoplasma biosensor that is sensitive to detecting human genomic DNA methylation levels of SOCS-1 in gastrointestinal tumors (56). By employing nanoimage printing technology, Gopal et al. successfully created netrin-1 nanodots with a specific distribution. The nanodots are capable of recruiting and aggregating the missing (dCC) receptor as well as F-actin in colorectal cancer, thereby providing new insights into the diagnosis and treatment of colorectal cancer (57). The use of carbon nanotubes in the diagnosis and treatment of colorectal cancer is a significant advancement due to their versatility. Abdolahad et al. successfully developed a novel electroendoscopic spectroscopic analysis tool utilizing vertically aligned carbon nanotubes (VACNTs) as the core component. This tool is capable of accurately distinguishing between the different cancerous stages of colorectal cancer by taking advantage of the electrical and optical properties of VACNTs (58). It has been established that membrane-bound TRAIL induces a more pronounced receptor aggregation and apoptosis than soluble TRAIL (59). In a recent study, Zakaria et al. demonstrated that nanovectorization of the tumor necrosis factor-associated apoptosis-inducing ligand TRAIL using single-walled carbon nanotubes significantly improves the efficacy of colorectal cancer tumor cell eradication (60). In addition to developing a novel tool for electroendoscopic spectroscopic analysis, Abdolahad has also fabricated a bioelectronic device based on silicon nanograss. This device is capable of detecting several human colon invasive cancer cells (SW48) in mixed cell cultures without any biochemical labeling of primary colon cancer cells (HT29). Furthermore, it is suitable for more accurate cancer staging (61). In a previous study, Wen et al. prepared a camouflaged ultrasound sensitizer by encapsulating hematoporphyrin molecules in a polylactic acid (PLA) matrix and subsequently coating them with cancer cell membranes (CCM) derived from colon tumor 26 (CT26) cells. This produced the H@PLA@CCM nanosystem. The biocompatible ultrasound nanosensitizer H@PLA@CCM, camouflaged with cancer cell membranes, has been shown to induce significant apoptosis and necrosis of tumor cells through effective sonodynamic therapy (SDT), as well as highly efficient acoustic kinetic ablation of colon tumors, thereby achieving tumor suppression (62). Wang et al. employed a combination of highly sensitive platinum-functionalized nanomaterials and noncontact hPICM to achieve precise measurement of H_2_O_2_ gradients from extracellular to intracellular compartments within individual colorectal cancer Caco-2 cells. This technique facilitates ion current measurements with high sensitivity and spatial resolution (38) (**Figure 3**).

In summary, the significance of nanomaterials in imaging technology is demonstrated by several factors, including the enhancement of imaging quality, the advancement of innovative imaging techniques, the expansion of imaging applications, and a deeper understanding of nanomaterial behavior in biological systems.

### Nanovaccines

Nanovaccines have the potential to be used not only for the prevention of colorectal cancer but also for its treatment and diagnosis. The insertion of cholesterol-modified CpG oligodeoxynucleotides (Chol-CPG), a Toll-like receptor 9 agonist, into the nuclear membrane of autologously derived *Fusobacterium nucleatum* by Chen et al. represents a promising avenue of research in this field. Similarly, the development of a safe and efficient bacterial vaccine (LipoFM-CPG) by Chen L, Kang Z, Shen J et al. is a significant step forward. The bacterial vaccine was capable of selectively preventing *F. nucleatum* infection over the long term by enhancing antigen presentation and inducing an immune response. This resulted in an improvement in the therapeutic efficacy of chemotherapy and a reduction in cancer metastasis in *F. nucleatum*-infected CRCs (63). Huang et al. employed a two-step emulsification method to synthesize poly(lactic-co-glycolic acid)/GA nanoparticles (PLGA/GA NPs). They then utilized CT26 colon CCM to develop nanovaccines, designated as CCM-PLGA/GA NPs. These nanoparticles were shown to possess a dual capacity: directly targeting and killing tumors by enhancing the tumor-targeting ability of GA, and indirectly promoting tumor cell death by activating dendritic cell maturation, thereby modulating the tumor immune microenvironment (64). It was demonstrated that cellular nanodiscs manufactured from cancer cell membranes and combined with lipid-based adjuvants for antitumor vaccination were effective in inhibiting the growth of colorectal cancer in mice (65).

**Figure 4:**
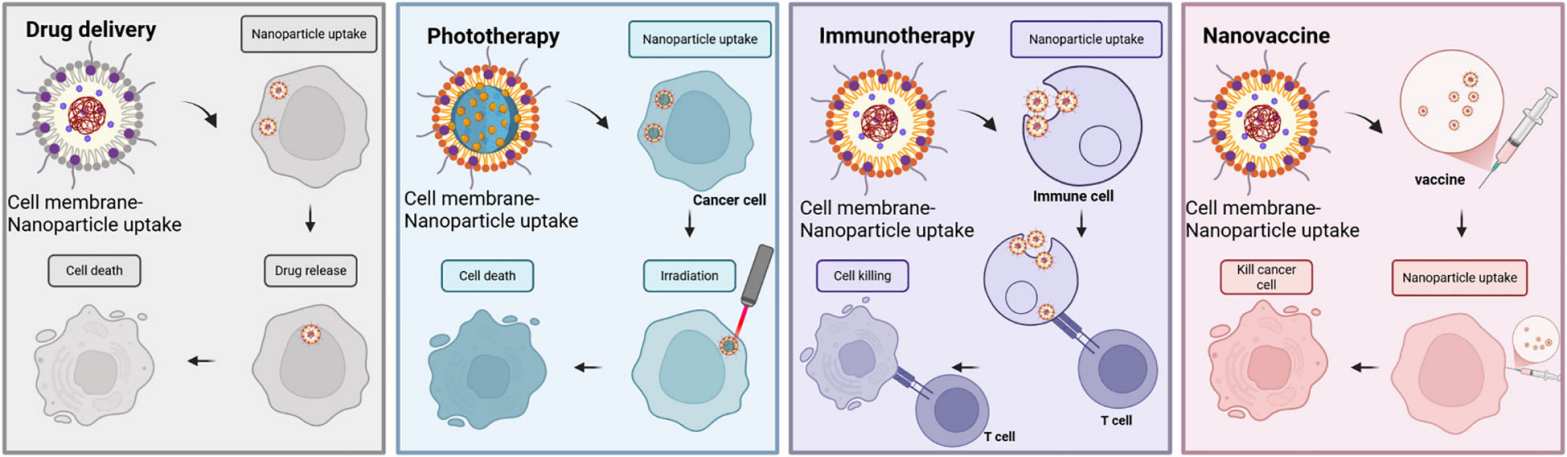
Anticancer applications of cell membrane-encapsulated nanoparticles. In cancer drug delivery, coatings derived from erythrocyte and cancer cell membranes enhance therapeutic efficacy by prolonging circulation and enabling targeted localization to tumors. Similarly, in phototherapy applications, cell membrane-encapsulated nanoparticles serve as excellent carriers for targeted delivery of photothermophiles and photosensitizers to tumors, thereby enhancing their effects under irradiation. In immunotherapy, cell membrane-encapsulated nanoparticles can deliver immunostimulants, either as a source of antigen or in direct contact with immune cells, to promote antitumor immunity. Taking advantage of their nanoscale size, nanovaccines have shown remarkable results against tumor cells. They play a unique role through efficient antigen delivery, precise targeting of tumor cells, and activation of a broad immune response.

To sum up, nanovaccines can be used not only to specifically target and kill cancer cells but also to label and monitor pathogens (**Figure 4**).

## Clinical research and challenges

In recent years, cell membrane-coated nanomaterials have been extensively investigated and have demonstrated remarkable targeting efficiency. However, their translation from laboratory research to clinical application remains challenging, primarily due to concerns regarding biodegradability, potential toxicity, and large-scale manufacturing costs. First, the biodegradability of CNPs is critical for clinical applications, and further research is needed to ensure that their degradation products are safe and do not pose long-term accumulation risks. Secondly, although CNPs exhibit low toxicity, their safety must be confirmed through long-term, large-scale clinical trials. One clinical trial (NCT06048367) is evaluating the safety and tolerability of intratumoral injections of carbon nanoparticle-loaded iron (CNSI-Fe[II]) in patients with advanced solid tumors.

The selection of appropriate nanomaterials is crucial, and U.S. Food and Drug Administration (FDA)-approved biodegradable nanoparticles, such as PLGA, should be prioritized to ensure the production of safe, biocompatible, and low-toxicity nanoparticles (66). Several FDA-approved nanomaterials are available for drug delivery platforms, and utilizing these materials can expedite the transition to clinical applications compared with unapproved alternatives (67).

The same consideration applies to the selection of animal models, which vary significantly in their ability to replicate human tumor physiology and immune responses. These differences can affect the clinical relevance of the findings and the feasibility of their translational application (68, 69). The human immune system is extremely complex, and no animal model can fully replicate all aspects of it. Therefore, the development and selection of appropriate models are crucial to ensure the successful clinical translation of research findings.

Although the transition from the laboratory to the clinic is challenging, several clinical trials are currently underway. As chemotherapeutic agents are highly toxic to both healthy and cancerous areas, effective targeting could provide substantial benefits for patients with advanced or metastatic tumors. For example, polymeric nanoparticles containing cetuximab, modified with growth inhibitor analogs, are being investigated for targeting colorectal cancer (NCT03774680). Clinical trials are evaluating the efficacy of oral nanoformulations of curcumin as an adjuvant therapy in the treatment of metastatic colorectal cancer with XELOX or FOLFOX regimens (IRCT20200408046990N7) (70). Clinicians are also investigating carbon nanoparticles as a lymph node tracer in laparoscopic colorectal surgery to improve lymph node sampling and detect micrometastases (NCT03350945) (71).

## Conclusion

In this review, we provide a comprehensive overview of the development and application of CNPs in colorectal cancer, highlighting how the type of membrane encapsulation influences their functionality (**Table 1**). The multifunctionality of CNPs offers significant potential for a range of therapeutic applications, including drug delivery, anti-inflammatory effects, photothermal therapy, and immunotherapy. Despite the challenges that remain for clinical translation, it is clear that cell membrane-encapsulated nanomaterials hold considerable promise as a tool for cancer immunotherapy. This approach is expected to enhance the efficacy of oncology treatments and contribute to the advancement of precision, personalized medicine.

**Table 1:** Tumor targeting using cell membrane-encapsulated nanoparticles.

Mode of action	Name	Membrane composition	Mechanism of action	Effect	
Drug delivery	cp-MΦ-NPs	Macrophage	Bind and neutralize proinflammatory cytokines	Reduce	(27)
	TGZ@eM	Erythrocyte	Aids in the delivery of GO*_x_* to tumor cells by starving the tumor of glucose and O_2_.	Inhibit	(33)
	RSV-NPs@RBCm	Erythrocyte	Enhances resveratrol’s ability to penetrate tumors by binding to the tumor-penetrating peptide iRGD	Inhibit	(40)
	M@NPs/miR21	Tumor cell and macrophage	Inhibits miR21 expression and induces apoptosis in tumor cells. Regulates Bcl-2 and Ki67 expression	Inhibit	(47)
Combined photothermal therapy (CT)	MPB-3BP@CM NPs	Tumor cell	Polarization of TAMs toward the M1 phenotype by blocking CD47-SIRPα interaction	Inhibit	(48)
	Fe_3_O_4_@PDA@CaCO_3_-ICG@CM	Tumor cell	Eliminate PD-L1 and TGF-β and enhance antitumor immune response	Inhibit	(50)
	GCMNPs	Leucocyte	Enhances antigen presentation and induces immunogenicity by blocking PD-1/PD-L1-activated T cells	Inhibit	(51)
	PDA/GNS@aPD-L1 NPs		Promote DC maturation and increase CD8^+^ T-cell infiltration. Enhance antitumor immune response	Inhibit	(52)
	GNC-Gal@CMaP		Activation of APCs enhances the efficacy of anti-PDL1 and galunisertib. It also increases antitumor immunity.	Inhibit	(54)
Combined acoustic dynamics (physics)	H@PLA@CCM	Tumor cell	Induces tumor cell apoptosis and necrosis	Inhibit	(62)
Nanovaccines	LipoFM-CPG	Bacterial	Enhance antigen presentation and induce an immune response	Inhibit	(63)
	CCM-PLGA/GA NPs	Tumor cell	Activating DCs to mature and modulating the tumor immune microenvironment indirectly kills cells	Inhibit	(64)
